# Apparent spontaneous clearance of chronic hepatitis C virus infection in a HIV co-infected patient with decompensated cirrhosis: a case report

**DOI:** 10.1097/QAD.0000000000000621

**Published:** 2015-05-06

**Authors:** Borja Mora-Peris, Robert D. Goldin, David Muir, Janice Main, Ricky Gellissen, Anthony Brown, Eleanor Barnes, Graham Cooke

**Affiliations:** a Department of Medicine, Faculty of Medicine, Imperial College London, St Mary's Hospital Campus, Norfolk Place; b Department of HIV and Genitourinary Medicine; c Department of Cellular Pathology, Imperial College Healthcare NHS Trust, St Mary's Hospital; d Department of Infection & Immunity, Imperial College Healthcare NHS Trust, Charing Cross Hospital, London; e Peter Medawar Building for Pathogen Research, Oxford University; f NIHR Oxford Biomedical Research Centre, Oxford, UK.

Spontaneous clearance of chronic hepatitis C virus (HCV) infection is unusual, once chronic disease is established, particularly in HIV/HCV co-infected individuals [1]. In cirrhotic patients, pegylated interferon-based regimens achieve lower sustained virological response (SVR) rates and increase the likelihood of decompensation [2]. However, interferon-sparing regimens using novel direct-acting antivirals (DAAs) are now becoming available and have dramatically increased the SVR rates among cirrhotic patients, minimizing the risk of hepatic decompensation [3,4].

We report a patient with HIV/HCV co-infection and decompensated cirrhosis who appeared to have cleared HCV spontaneously on intensive investigation and, therefore, was not offered interferon-free treatment.

A 43-year-old heterosexual man with a history of prior intravenous drug use was initially diagnosed with HIV-1 infection in 2008 in the context of *Pneumocystis jirovecii* pneumonia. At baseline, he had a CD4^+^ cell count of 30 cells/μl, plasma HIV RNA of 39 911 copies/ml, positive anti-HCV (Abbott Architect; Abbott Diagnostics, Abbott Park, Illinois, USA) and confirmed HCV genotype 4 infection by sequencing (Micropathology Ltd, Coventry, UK) with a plasma HCV RNA level of 602 745 IU/ml (Abbott Real Time HCV PCR, M2000 system; Abbott Diagnostics). He was later shown to be IL28B-CC genotype. Screening demonstrated past hepatitis B virus (HBV) infection with negative surface antigen and undetectable HBV DNA. Combination antiretroviral therapy (cART) with emtricitabine/tenofovir/efavirenz (200/300/600 mg) once daily was initiated shortly after HIV diagnosis and, by 1 year of treatment, his CD4^+^ cell count had improved to 310 cells/μl. Soon afterwards, he developed decompensation of his chronic liver infection in the form of ascites, jaundice and mild encephalopathy (Child–Pugh B). A liver biopsy showed florid periportal bile duct reaction, marked lobular inflammation and fibrosis scoring 4/6. A decision for HCV treatment was deferred whilst the patient attempted to reduce his high BMI. Regular alpha-fetoprotein levels, liver ultrasound and MRI scans were performed without evidence of hepatocellular carcinoma.

His cirrhosis progressed to Child–Pugh C and the patient was referred for liver transplantation, which was turned down due to a low predicted 5-year survival, an assessment influenced by the patient's refusal to receive blood products according to his beliefs as a Jehovah's witness.

His liver function deteriorated further despite medical management. To minimize drug interactions in case of transplantation and/or HCV treatment, efavirenz was switched to raltegravir 400 mg twice daily in January 2013. Around that time, plasma HCV RNA levels experienced a downward trend, achieving values of less than 15 HCV RNA IU/ml (Roche Cobas TaqMan; Roche Molecular Diagnostics, Pleasanton, California, USA) in January 2014. At that time, he was considered for early access to DAA therapy (sofosbuvir with either ledipasvir or daclatasvir). Subsequently, no HCV RNA was detected on three occasions (February, April and June 2014). In parallel, CD4^+^ cell count increased from 370 cells/ml (21%) in April 2012, to 955 cells/ml (31%) in June 2014 (Fig. 1).

**Fig. 1:**
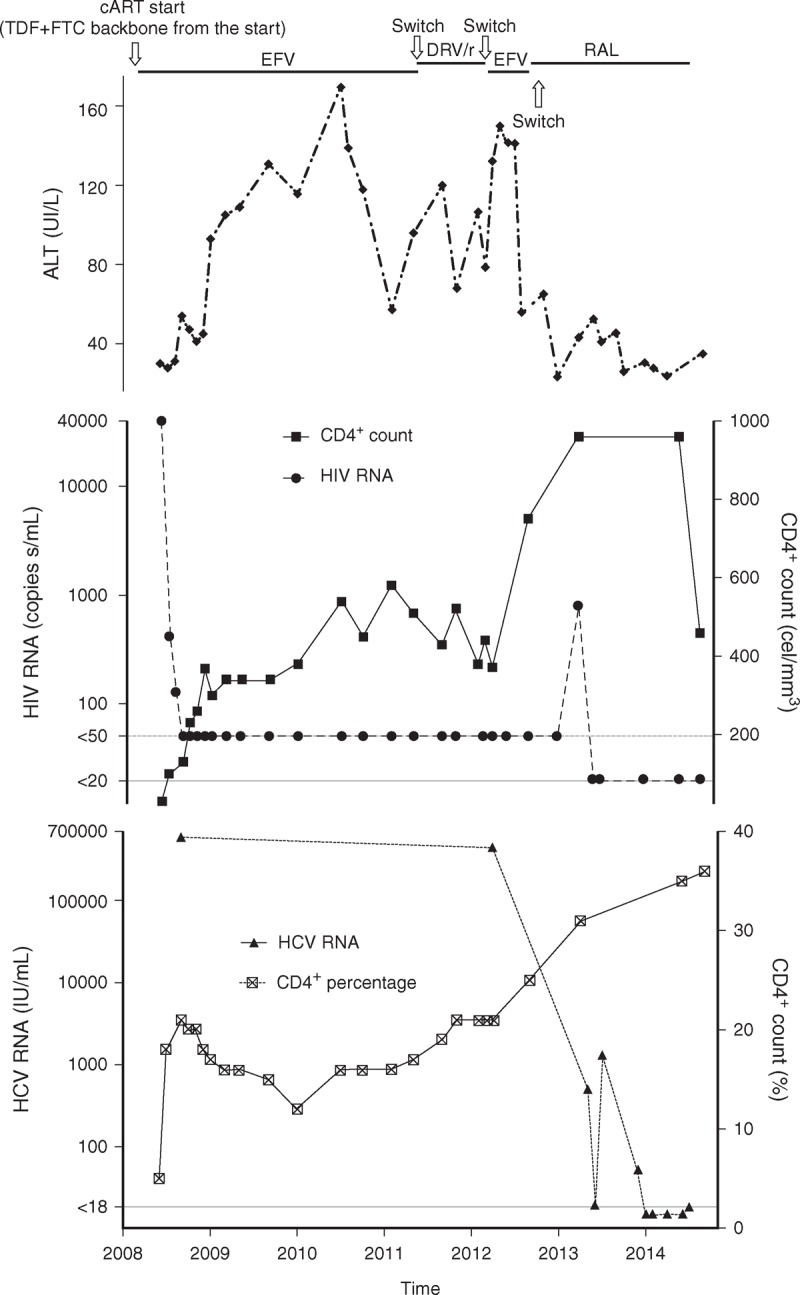
Time trends from HIV presentation in May 2008 for antiretroviral regimes (with fixed nuke-backbone maintained throughout regimes) and laboratory results: ALT, HCV RNA, HIV RNA, absolute CD4^+^ cell count and CD4^+^ percentage.

ALT, alanine transaminase; cART, combined antiretroviral therapy; DRV/r, darunavir/ritonavir; EFV, efavirenz; FTC, emtricitabine; HCV, hepatitis C virus; RAL, raltegravir; RNA, ribonucleic acid; and TDF, tenofovir.

A transjugular liver biopsy was performed showing evidence of inactive cirrhosis, mild portal and nodular inflammation, no large cell or fatty change, negative stains for iron and alpha-1 antitrypsin consistent with resolved infection and no evidence of HCV RNA on PCR (Micropathology Ltd). An HCV-specific enzyme-linked ImmunoSpot that assessed interferon-γ production in peripheral blood mononuclear cells (stimulated by gt1 and gt4 HCV peptides) at this time was not reactive, suggesting recovery of CD8^+^ and CD4^+^ T-cell immunity was not responsible for his HCV clearance.

Spontaneous clearances of established HCV infection have been previously described in absence of specific anti-HCV CD4^+^ responses [5]. Although rare, HCV clearance among HIV–HCV co-infected patients on cART is mainly described in patients with IL-28B CC genotype, suggesting this subset of patients might benefit from an earlier initiation of cART [1,6–9]. The initial period of HCV undetectability coincided with a switch to raltegravir and a marked increase in peripheral CD4^+^ cell count. Whether this was causal is unclear. However, maintained HIV viral suppression [10] and improvements in drug-associated hepatotoxicity have been described in cirrhotic HCV–HIV co-infected patients after switching to raltegravir [11].

What makes this case more unusual is that, given the potential availability of novel DAAs to a limited number of patients, invasive liver investigation was undertaken, which not only failed to find virus, but also showed little evidence of active infection. Cases such as this one raise important implications for patients with very advanced disease being considered for DAA therapy. The potential for low-level viraemia, and even prolonged periods of aviraemia, is well recognized [12]. Whether in these patients more invasive investigations are helpful to exclude the possibility for a hidden focus of HCV infection is unclear. It remains uncertain as to whether, in the absence of viraemia, the likelihood of persistent viral reservoirs should be sufficient to justify treatment.

## Acknowledgements

The authors would like to thank the patient, as well as the nursing and pharmacy staff at the Department of HIV and Genitourinary Medicine at St Mary's Hospital (Imperial College Healthcare NHS Trust, London, UK). This work was supported in part by the BRC of Imperial College NHS Trust. EB and GC are supported are part of the MRC STOP HCV and NHIC consortia.

### Conflicts of interest

There are no conflicts of interest.
